# Characteristics of Heart Rate Tracings in Preterm Fetus

**DOI:** 10.3390/medicina57060528

**Published:** 2021-05-25

**Authors:** Maria F. Hurtado-Sánchez, David Pérez-Melero, Andrea Pinto-Ibáñez, Ernesto González-Mesa, Juan Mozas-Moreno, Alberto Puertas-Prieto

**Affiliations:** 1 Obstetrics and Gynecology Service, Virgen de las Nieves University Hospital, 18014 Granada, Spain; franihurs@gmail.com (M.F.H.-S.); apuertas51@hotmail.com (A.P.-P.); 2 Anesthesiology, Resuscitation and Pain Therapy Service, Virgen de las Nieves University Hospital, 18014 Granada, Spain; dpmelero@yahoo.es; 3 Obstetrics and Gynecology Service, Poniente Hospital, 04700 El Ejido (Almería), Spain; apintoib@gmail.com; 4 Obstetrics and Gynecology Service, Regional University Hospital of Malaga, 29011 Malaga, Spain; egonzalezmesa@gmail.com; 5 Department of Obstetrics and Gynecology, University of Granada, 18016 Granada, Spain; 6 Consortium for Biomedical Research in Epidemiology & Public Health (CIBER Epidemiología y Salud Pública-CIBERESP), 28029 Madrid, Spain; 7 Biohealth Research Institute (Instituto de Investigación Biosanitaria Ibs.GRANADA), 18014 Granada, Spain

**Keywords:** fetal heart rate, preterm fetus, cardiotocography, fetal heart rate patterns, electronic fetal monitoring

## Abstract

*Background and Objectives:* Prematurity is currently a serious public health issue worldwide, because of its high associated morbidity and mortality. Optimizing the management of these pregnancies is of high priority to improve perinatal outcomes. One tool frequently used to determine the degree of fetal wellbeing is cardiotocography (CTG). A review of the available literature on fetal heart rate (FHR) monitoring in preterm fetuses shows that studies are scarce, and the evidence thus far is unclear. The lack of reference standards for CTG patterns in preterm fetuses can lead to misinterpretation of the changes observed in electronic fetal monitoring (EFM). The aims of this narrative review were to summarize the most relevant concepts in the field of CTG interpretation in preterm fetuses, and to provide a practical approach that can be useful in clinical practice. *Materials and Methods:* A MEDLINE search was carried out, and the published articles thus identified were reviewed. *Results:* Compared to term fetuses, preterm fetuses have a slightly higher baseline FHR. Heart rate is faster in more immature fetuses, and variability is lower and increases in more mature fetuses. Transitory, low-amplitude decelerations are more frequent during the second trimester. Transitory increases in FHR are less frequent and become more frequent and increase in amplitude as gestational age increases. *Conclusions:* The main characteristics of FHR tracings changes as gestation proceeds, and it is of fundamental importance to be aware of these changes in order to correctly interpret CTG patterns in preterm fetuses.

## 1. Introduction

Cardiotocography (CTG) aims to identify fetal heart rate (FHR) patterns that may indicate a risk, so that clinicians can forestall problems with measures intended to improve perinatal outcomes. As a tool with high sensitivity but very low specificity, the false positive rate of CTG findings approaches 60%. This means that watchful waiting is advised when a CTG tracing is considered normal, whereas abnormal FHR tracings offer no certainties regarding the hypoxic status of the fetus or possible acidosis [1].

In an effort to determine the influence of CTG on perinatal outcomes, Alfiveric et al. updated a Cochrane review in 2017 [2] in order to evaluate the effectiveness and safety of continuous CTG as a form of electronic fetal monitoring (EFM) for fetal assessment during labor, compared to intermittent monitoring. The authors concluded that CTG during labor is associated with reduced rates of neonatal seizures, but no clear differences in cerebral palsy, infant mortality or other standard measures of neonatal wellbeing. However, continuous CTG was associated with an increase in cesarean deliveries and instrumental vaginal births.

In the particular case of preterm gestations, no clinical practice guidelines are currently available for EFM, and published studies on CTG in preterm fetuses are scarce. Many characteristics of CTG tracings depend on gestational age and reflect the degree of development and maturity of regulatory centers in the central nervous system and cardiovascular system. This makes it imperative to understand these physiological characteristics in order to correctly interpret FHR patterns in preterm fetuses [3].

Because they are less developed and less mature, preterm fetuses may respond to stress in anomalous ways, giving rise to situations of permanent hypoxia in the fetal brain at threshold values that may be lower than in term fetuses [4]. As a result, the classic characteristics of CTG tracings in healthy term fetuses exposed to hypoxic situations may not be observable in preterm fetuses.

The FHR is regulated by the autonomous nervous system, and during fetal development the sympathetic nervous system develops much earlier than the parasympathetic system, which develops mainly throughout the third trimester. The sympathetic nervous system is activated in situations of stress; accordingly, preterm fetuses will have a higher baseline FHR with an apparent reduction in variability, owing to the predominant action of the sympathetic nervous system and lesser opposition from the parasympathetic system [3].

The evidence suggests that non-reassuring CTG tracings are of greater importance for adverse neonatal outcomes in preterm fetuses than in term fetuses. In this regard, Freeman et al. [5] claimed that 20% of term fetuses have signs of neurological depression at birth associated with non-reassuring CTG patterns, compared to 70–80% of preterm fetuses with the same type of FHR tracings. These authors sustained that preterm fetuses are more susceptible to hypoxic damage and have a greater tendency to develop prematurity-related complications if they are born with hypoxia or acidosis. In addition, they claimed that the change from reassuring to non-reassuring CTG features occurs more frequently and faster in preterm fetuses than term fetuses.

Another study by Matsuda et al. [6] analyzed the critical period of non-reassuring FHR patterns in preterm gestation. Of a total of 772 preterm deliveries, they observed non-reassuring FHR patterns in 181 (23.4%), consisting of recurrent late decelerations with loss of variability, prolonged decelerations, severe recurrent variable decelerations, and recurrent late decelerations. Umbilical cord artery blood pH in cases of recurrent late decelerations with loss of variability (7.15 ± 0.11, *p* < 0.01) and prolonged decelerations (7.17 ± 0.16, *p* < 0.01) was significantly lower than in the group with reassuring FHR patterns (7.29 ± 0.06). Fetal acidosis was more frequent in fetuses with recurrent late decelerations with loss of variability (24.1%) and prolonged decelerations (22.9%) than in those with other non-reassuring FHR patterns or with reassuring FHR patterns (2.9%). The largest differences compared to term fetuses were that preterm fetuses sometimes developed abnormal FHR patterns rapidly, and these patterns sometimes became more serious much more rapidly than in term fetuses.

The aims of this narrative review were to summarize the most relevant concepts in the field of interpretation of CTG tracings in preterm fetuses, and to provide a practical approach that can be useful in clinical practice.

## 2. Materials and Methods

**Figure 1:**
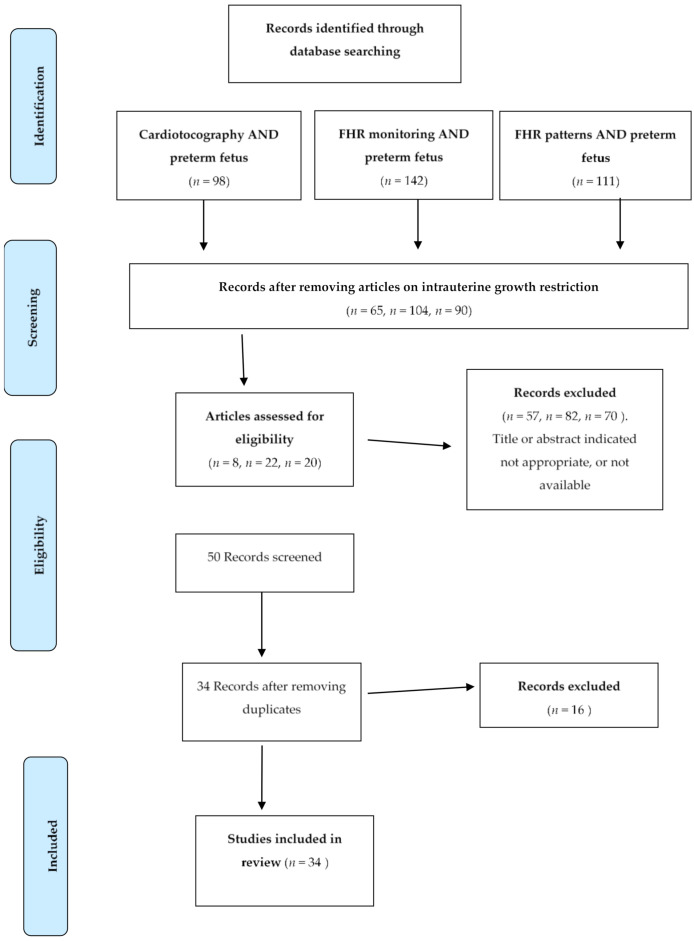
Flow diagram of identification of eligible studies. FHR: fetal heart rate.

A MEDLINE search was carried out covering the period from 1980 until 2020. Searches with the terms “cardiotocography AND preterm fetus” identified 98 published studies. After studies of fetuses with intrauterine growth restriction (IGR) were removed, 65 publications remained. A further 57 studies were excluded as being outside the scope and aims of the present review, i.e., to analyze the main characteristics of CTG in preterm fetuses. Searches with the terms “electronic FHR monitoring AND preterm fetus” identified 142 publications; after excluding those that centered on fetuses with IGR, 104 studies remained, and after excluding those that did not comply with our review criteria, 22 remained. A third search with the terms “FHR patterns AND preterm fetus” located 111 published items, of which 90 remained in our sample after those focusing on fetuses with IGR were excluded. After excluding those that did not comply with our review criteria, 20 remained. The pooled results of the three searches yielded a total of 50 publications, of which a further 16 were excluded as duplicates. The final sample consisted of 34 studies, 7 of which had been published within the previous 10 years (Figure 1).

## 3. Results and Discussion

### 3.1. Baseline FHR

Classic studies show that FHR increases steadily until week 16 of gestation and decreases thereafter by one beat per minute (bpm) each week, as a result of maturation of the parasympathetic nervous system [7]. Preterm fetuses, with their less developed parasympathetic system, usually have a higher baseline FHR compared to term or post-term fetuses, with their more developed parasympathetic system.

There is broad consensus among most scientific societies (e.g., International Federation of Gynecology and Obstetrics (IFGO) and American College of Obstetricians and Gynecologists (ACOG)) and clinical practice guidelines (e.g., The National Institute for Health and Care Excellence (NICE) and The Society of Obstetricians and Gynaecologists of Canada (SOGC)) in considering the normal range of FHR to be 110–160 bpm, although occasionally a term fetus may have a baseline FHR between 90 and 110 bpm as a consequence of greater parasympathetic nervous system maturity. This can be considered normal if other parameters in the CTG tracing are reassuring. Nonetheless, some current thinking advocates a less stringent application of the criteria for normal findings generally used to interpret CTG tracings, given that some parameters used to evaluate them may be generalized without due consideration for the particular characteristics of each fetus, e.g., gestational age [8].

As gestational age progresses, maturation of the parasympathetic nervous system leads to a decrease in baseline FHR, although it generally remains within the normal range of 110 to 160 bpm. In preterm fetuses, baseline FHR is close to the generally accepted upper limit of normality, and as noted, decreases as gestation proceeds [9]. Shuffrey et al. [10] published the largest longitudinal study carried out to date to analyze changes in FHR, variability, and fetal body movements throughout gestation in healthy fetuses. This study monitored 1655 fetuses during Weeks 20–24 (GA1), Weeks 28–32 (GA2), and Weeks 34–38 (GA3). The authors found that FHR decreased as gestation progressed, with mean rates of 144.1 ± 4.8 bpm in GA1, 139.5 ± 5.7 bpm in GA2, and 136.4 ± 6.7 bpm in GA3. These results were consistent with earlier studies [11,12,13,14,15,16] such as the one published by Amorim-Costa et al. [11], who followed 145 cases and also reported decreasing FHR as gestation progressed, with a mean rate of 143.7 bpm at 24 weeks, 138.9–138.4 bpm at 28–32 weeks, and 135.5–135.4 bpm at 34–38 weeks.

The findings published in these studies are consistent with unpublished data for a population analyzed at Hospital Universitario Virgen de las Nieves (HUVN) in Granada (Spain) for a descriptive, observational study of CTG tracings in preterm fetuses with no gestational disorders or anomalies in the absence of uterine contractions (Table 1). The CTG tracings were studied for a total of 118 preterm fetuses between Weeks 22 and 36 of gestation. In 66 cases (55.9%) the fetus was younger than 30 weeks of gestation, and in remaining 52 cases (44.1%) comprised preterm fetuses of more 30 weeks of gestation. Mean baseline FHR was 141 ± 8 bpm. Only one fetus (0.8%) had a baseline FHR lower than 110 bpm; all other fetuses (99.2%) had an FHR within the commonly accepted normal range, i.e., between 110 and 160 bpm. Mean baseline FHR in the group of fetuses of less than 30 weeks of gestation was 144 ± 6 bpm, versus 138 ± 8 bpm (*p* < 0.05) in fetuses of 30 weeks’ gestation or more; in other words, the findings were consistent with currently available evidence.

In preterm fetuses, as in term fetuses, an FHR higher than 160 bpm is considered tachycardia. However, this event is more frequent in preterm fetuses and is more predictive of acidemia, low Apgar scores, and adverse neonatal outcomes compared to tachycardia observed in term fetuses [4].

The effects on FHR of certain drugs used in preterm gestations merit particular attention. In preterm fetuses, ritodrine causes fetal tachycardia as a result of β-adrenergic receptor stimulation, associated with decreased variability. In addition, corticosteroids are also related with increases in FHR [17].

### 3.2. Transitory FHR Accelerations (Reactivity)

Transitory FHR accelerations (reactivity) occur as a result of somatic activity by fetus, and are first apparent in the second trimester of gestation. As the fetus matures, the number of transitory FHR accelerations increases as the time elapsed between episodes decreases.

Controversy continues to surround the percentage of fetuses with reactivity at different gestational ages. Some research [18] found that at 24 weeks of gestation only 50% of healthy fetuses showed transitory FHR accelerations, and that at 30 weeks of gestation these accelerations were seen in more than 95%. In contrast, other studies [16,19,20] claimed that between Weeks 20 and 30 only 13% of fetuses showed no transitory FHR increases over a period of 60 min, and that between Weeks 24 and 32, fetal respiratory movements and the number of fetal gross body movements increased. These studies concluded that 20% of transitory FHR accelerations were unrelated to fetal movements, and that 30% of fetal movements were not associated with transitory FHR increases in fetuses of less than 32 weeks’ gestation.

Data from the HUVN in Granada (Table 1) show that 75.4% of preterm fetuses between 32 and 36 weeks of gestation had transitory FHR increases, and 53.4% of the CTG tracings showed reactivity, i.e., at least two transitory increases greater than 10 bpm and longer than 10 s over a 20-min period. Of these increases, 47.6% were seen in CTG tracings for fetuses of <30 weeks’ gestation and 52.4% were seen in fetuses of ≥30 weeks, with no statistically significant difference between gestational age groups.

The National Institute of Child Health and Human Development [21] and later the ACOG [22] defined transitory FHR increases in gestations of less than 32 weeks as increases of ≥10 bpm above baseline during ≥10 s. The distinction between gestations of less than 32 weeks vs. the classic definition of ≥15 bpm during ≥15 s in gestations of ≥32 weeks was justified on the basis of changes seen in FHR as the autonomous nervous system matures. Despite widespread acceptance that the amplitude of transitory increases is lower in preterm fetuses than in term fetuses, to date there have been no well-designed trials that support this assumption or that establish the safety of this claim [23].

In light of these findings, it is generally accepted that before 32 weeks of gestation the frequency and amplitude of FHR accelerations are lower. Accordingly, before this gestational age the CTG tracing can be considered indicative of reactivity if it shows two FHR accelerations of at least 10 bpm above the baseline value, and if the accelerations last at least 10 s (10 × 10 criterion).

The validity of the 10 × 10 criterion before 32 weeks of gestation has been analyzed in two studies. In a sample of 143 pregnancies [23], groups matched for other characteristics were compared according to the 10 × 10 reactivity criterion (*n* = 72) vs. the 15 × 15 criterion (*n* = 73). The frequency of adverse perinatal outcomes was similar when CTG reactivity was defined with the 10 × 10 criterion rather than the conventional 15 × 15 criterion. However, the statistical significance was insufficient to confirm the safety of using the 10 × 10 criterion before 32 weeks of gestation. Another study of 488 pregnancies [24] used EFM to compare the relationship of the 10 × 10 vs. 15 × 15 evaluation criteria for FHR accelerations with perinatal outcomes. This analysis of 7100 CTG tracings found no association between either reactivity criterion and perinatal outcomes in terms of perinatal mortality, need for neonatal resuscitation, 5-mintute Apgar score less than 7, mechanical ventilation, or intraventricular hemorrhage. Current evidence is thus insufficient to dispel questions regarding the value of 10 × 10 transitory FHR accelerations in preterm fetuses that were previously found to be mature enough to generate 15-bpm accelerations during 15 s (15 × 15).

Reactivity in preterm fetuses can be affected by a number of drugs. The findings with regard to magnesium sulfate are inconclusive, although most studies have reported a decrease in the frequency of transitory FHR accelerations [25]. A study of the effect of corticosteroids [17] found these drugs were able to increase fetal movements and FHR accelerations within the first 24 h after administration, followed by a reduction in both during the subsequent 96 h, although these changes did not imply fetal deterioration.

### 3.3. Decelerations in FHR

In fetuses between 20 and 30 weeks of gestation the CTG tracing usually discloses FHR decelerations in the absence of uterine contractions. These brief, low-amplitude decelerations frequently appear in intrapartum CTG tracings, as reported by Sorokin et al. [7].

Variable decelerations are more frequent in preterm fetuses both antepartum and intrapartum; in the latter period these decelerations appear in 70–75% of all preterm fetuses compared to 30–50% of term fetuses. These changes are believed to be related with the smaller amount of Wharton’s jelly in the umbilical cord and greater contractile force of the heart owing to the lesser degree of development of the fetal myocardium. Variable intrapartum decelerations are associated with a higher rate of hypoxemia, acidemia, abnormal neurological processes, and adverse neonatal outcomes in the long term. Current evidence suggests a relationship with intraventricular hemorrhaging via a mechanism independent of fetal acidemia [3].

The same trend in variable decelerations was seen in the antepartum CTG data for preterm fetuses analyzed at HUVN in Granada (Table 1). At least one typical variable deceleration was seen in 5.6% of the cases, and these alterations were seen periodically in 3.2% of the cases. The differences between gestational age groups were significant: decelerations were seen in 85.7% of fetuses at <30 weeks compared to 14.3% of fetuses at ≥30 weeks (*p* < 0.05).

The frequency of late decelerations was similar in preterm and term fetuses, although obstetric conditions associated with this type of deceleration were more frequent in the former group (uteroplacental insufficiency, intra-amniotic infection, preeclampsia, IGR, and *abruptio placentae*). Late decelerations are of more concern in preterm fetuses because of their association with hypoxemia, acidemia and abnormal neurological processes in the long term. In this connection, Matsuda et al. [6] found that the FHR features associated most frequently with low cord arterial blood pH at birth were late decelerations accompanied by decreased variability, and prolonged decelerations.

### 3.4. FHR Variability

Low or absent variability (<5 bpm) is more frequent in preterm fetuses. Research by Shuffrey et al. [10] confirmed that mean FHR variability increases during gestation from 2.9 ± 0.7 bpm at 24–26 weeks to 3.7 ± 0.9 bpm at 28–32 weeks, and 4.3 ± 0.1 bpm at 34–36 weeks. Among the preterm fetuses analyzed at our center (Table 1), 16.1% had minimal variability, and 82.2% had moderate variability (between 6 and 25 bpm). In the group of fetuses with a gestational age < 30 weeks, 57.9% showed diminished variability compared to 42.1% of fetuses in the group of ≥30 weeks’ gestation, whereas moderate variability was seen in 55.7% and 44.3% of the fetuses, respectively, in these two groups, although the differences between groups were not statistically significant.

An indicator of fetal wellbeing is the presence of cyclic FHR patterns, with periods of activity characterized by increased variability alternating with periods of apparently decreased variability. As gestational age increases, FHR patterns tend to stabilize [18]. Consequently, in very premature fetuses these cyclic features may not be present, owing to functional immaturity of the central nervous system rather than to the effect of hypoxia.

Signs of FHR variability in CTG tracings are also affected by certain drugs. For example, biophysical activities in fetuses are depressed after the administration of betamethasone to favor pulmonary maturation. In this connection, Mulder et al. [26] found a significant decrease in FHR variability after the administration of this drug, and their findings are consistent with observations from a randomized study by Senat et al. [27], who found that after betamethasone treatment was suspended, FHR variability returned to pretreatment values within one week. These authors found no differences in FHR after the administration of dexamethasone for fetal pulmonary maturation. However, Rotmensch et al. [28] reported decreased FHR variability after treatment with both drugs, and noted the effect was more pronounced with betamethasone than with dexamethasone. Regarding other drugs, magnesium sulfate has been associated with a lower FHR, decreased variability, and fewer prolonged decelerations, albeit with no evidence of adverse effects on neonatal outcomes [29].

A systematic summary of the findings regarding the relationship between FHR signal features and the degree of prematurity is presented in Table 2.

## 4. Conclusions

Despite the absence of standards for determining which characteristics of CTG tracings or FHR patterns can be considered unequivocally normal for preterm fetuses, what little evidence has accumulated to date shows clear differences in FHR patterns between preterm and term fetuses, and these differences must be taken into account to ensure that the patterns are evaluated appropriately. In summary, the four main features of FHR patterns in preterm fetuses compared to term fetuses are: (1) slightly higher baseline FHR, with increasing immaturity associated with increasing FHR (although the normal range remains 110 to 160 bpm), (2) lower variability, which increases as the fetus matures, (3) more frequent appearance of transitory, low-amplitude decelerations in the second trimester of gestation, and (4) lower frequency of transitory FHR increases, which become more frequent and increase in amplitude as gestational age increases.

All this information from CGT tracings in preterm fetuses must be transferred to clinical decision making. In this sense, it can be stated that (1) FHR of the preterm fetuses must be assessed according to its gestational age, (2) the assessment should be made taking into account whether the pregnancy is accompanied by pathology and the possible influence of the drugs administered to the pregnant woman, (3) fetal damage after hypoxic situations is earlier in the preterm than at term fetuses, so clinical decisions must take this variable into account, and (4) before accepting that deviations from normality of the FHR are due only to prematurity, it must be taken into account that their origin may be in a concomitant fetal pathological situation.

## Figures and Tables

**Table 1:** Cardiotocographic tracings in 118 preterm fetuses at Virgen de las Nieves University Hospital, Granada, Spain.

	<30 Weeks	≥30 Weeks	Total	*p*
*n*	66 (55.9%)	52 (44.1%)	118 (100%)	
Baseline FHR (bpm)	144 ± 6	138 ± 8	141 ± 8	<0.05
Minimal variability (≤5 bpm) *n* (%)	11 (57.9%)	8 (42.1%)	19 (16.1%)	ns
Moderate variability (6–25 bpm) *n* (%)	54 (55.7%)	43 (44.3%)	97 (82.2%)	ns
Reactivity *	30 (47.6%)	33 (52.4%)	63 (53.4%)	ns
Decelerations	14 (85.7%)	3 (14.3%)	17 (14.4%)	<0.05

* Presence of ≥2 transitory increases in FHR (fetal heart rate) at least 10 bpm in amplitude and at least 10 s in duration within a 20-min period. *n* (%): number (percentage); ns: not significant.

**Table 2:** Systematic summary of the findings regarding the relationship between FHR signal features and the degree of prematurity.

	<26 Weeks	26–32 Weeks	>32 Weeks
Baseline FHR (mean bpm)	144	139	136
Reactivity (transitory FHR accelerations)	May not be present or very reduced	Progressively increases	Similar to term fetuses
Decelerations	Common antepartum and intrapartum	Variable decelerations less frequent	Variable decelerations more frequent intrapartum
Variability	Reduced	Reaches normal >30 weeks	Similar to term fetuses

## Data Availability

No new data were created or analyzed in this study. Data sharing is not applicable to this article.
